# Anthropomorphism of Robots: Study of Appearance and Agency

**DOI:** 10.2196/12629

**Published:** 2019-05-10

**Authors:** Charles R Crowell, Jason C Deska, Michael Villano, Julaine Zenk, John T Roddy Jr

**Affiliations:** 1 Department of Psychology University of Notre Dame Notre Dame, IN United States; 2 Department of Psychology University of Toronto Toronto, ON Canada

**Keywords:** psychology, social, social perception, theory of mind, cognitive science, perception, cognition, robotics, telerobotics, human factors engineering

## Abstract

**Background:**

As the prevalence of robots increases each year, understanding how we anthropomorphize and interact with them is extremely important. The three-factor theory of anthropomorphism, called the Sociality, Effectance, Elicited agent Knowledge model, guided this study. As anthropomorphism involves a person making attributions of human likeness toward a nonhuman object, this model implies that anthropomorphism can be influenced either by factors related to the person or the object.

**Objective:**

The aim of this study was to explore factors influencing the anthropomorphism of robots, specifically the robot’s appearance (humanoid vs nonhumanoid) and agency (autonomous vs nonautonomous). We expected a humanoid robot would be anthropomorphized to a greater extent than one that was nonhumanoid. In addition, we expected that inducing an agency belief to the effect that a robot was making its own decisions would increase anthropomorphism compared with a nonagency belief that the robot was being remotely controlled by a human. We also sought to identify any role gender might play in anthropomorphizing the robot.

**Methods:**

Participants (N=99) were primed for agency or nonagency belief conditions and then saw a brief video depicting either a humanoid or nonhumanoid robot interacting with a confederate. After viewing the video, they completed 4 measures: perception to humanoid robots scale (PERNOD), the Epley anthropomorphic adjectives measure, the Fussel anthropomorphic adjective checklist, and the Anthropomorphic Tendencies Scale (ATS).

**Results:**

Findings with the PERNOD scale indicated subjects did perceive the 2 robots differently, *F*_6,86_=6.59, *P*<.001, which means the appearance manipulation was effective. Results with the Epley adjectives indicated that participants were more willing to attribute humanlike behavioral traits to the nonhumanoid rather than the humanoid robot, *F*_1,91_=5.76, *P*=.02. The Fussel adjective checklist results showed that subjects were more willing to attribute humanlike social qualities to the remote controlled than the autonomous robot, *F*_1,91_=5.30, *P*=.02. Finally, the ATS revealed the only gender effects in this study, with females reporting more endorsement of anthropomorphism for pets (*P*=.02) and less for showing negative emotions toward anthropomorphized objects (*P*<.001) if they had witnessed the humanoid rather than the nonhumanoid robot.

**Conclusions:**

Contrary to our expectations, participants were less willing to make humanlike attributions toward a robot when its morphology was more humanlike and were more willing to make those attributions when they were told that the robot was being remotely controlled by a person rather than acting on its own. In retrospect, these outcomes may have occurred because the humanoid robot used here had a smaller overall stature than the nonhumanoid robot, perhaps making it seem more toylike and because subjects made attributions toward the person behind the remote-controlled robot rather than toward the robot itself.

## Introduction

### Anthropomorphism and the Sociality, Effectance, Elicited Agent Knowledge Model

Anthropomorphism can be defined as “the tendency to imbue the real or imagined behavior of nonhuman agents with human like characteristics, motivations, intentions, or emotions” [1]. Instances of anthropomorphism occur all around us on a daily basis, from the tendency to imbue pets with human like traits [2] to the attribution of human like characteristics to deities [1] and even to personal computers [3]. As computers and robots become increasingly ubiquitous, our understanding of how we anthropomorphize robots in human robot interactions (HRI) will become more important as well [4].

### The Sociality, Effectance, Elicited Agent Knowledge Model of Anthropomorphism

Epley et al [1,2] have proposed a 3-factor theory, called the Sociality, Effectance, Elicited agent Knowledge (SEEK) model, to explain why human beings anthropomorphize. The first factor in the SEEK model is *sociality motivation*, or the human desire for social connections [1]. Humans are social animals with a strong need to establish and maintain a sense of interpersonal connection to others. Sociality motivation increases the tendency to search actively for sources of social connections in one’s environment or to invent those connections when necessary.

The second SEEK factor is *effectance motivation*, or the need to understand, control, and interact effectively with the environment [1]. This factor can give rise to the desire to understand the behavior of nonhuman agents by projecting onto them more familiar human traits. In this way, anthropomorphism serves as a tool to facilitate understanding of (and potentially control over) unfamiliar agents by making them more humanlike. This tendency can be exacerbated in situations where the behavior of a nonhuman agent cannot be accounted for easily by other explanations. For example, researchers have reported the results of a study in which participants were shown a brief video clip of 2 dogs interacting with each other where 1 dog was more behaviorally unpredictable [2]. Results indicated that participants were more likely to ascribe human like qualities to the less predictable dog. This finding seems consistent with the possibility that attributions of human like agency to nonhuman entities, including robots, are more likely when alternative accounts of agent behavior are not readily available [5].

The third SEEK factor, *elicited agent knowledge*, refers to the extent to which people have, and can, apply relevant anthropocentric knowledge to objects or entities that might be targets for the attribution of human like qualities. Homocentric knowledge often serves as the basis for making inferences about lesser known, nonhuman agents [1]. It follows, then, that physical appearance and movement of a nonhuman agent might be important factors in guiding elicited agent knowledge. That is, the evocation and application of anthropocentric knowledge might be facilitated by the morphological and kinetic similarity between human and nonhuman agents [1,4,6]. Recent studies with robots consistent with this possibility has shown that greater robot human likeness affects the receptivity of humans to advice provided by a robot [7], the extent to which humans will empathize with a robot [8], how much credit a human will take in a joint human robot task for successful task completion [9], and even the types of tasks for which a robot is deemed suitable [10,11]. Furthermore, when a person believes a robot shares his or her own gender, that person is more likely to attribute a human mind to the robot [12].

### Gender Differences in Human Robot Interaction and Anthropomorphism

Another factor potentially influencing the tendency of humans to anthropomorphize robots is gender. Several studies have explored how gender affects HRI. One study showed that males and females provided significantly different answers to social desirability questions asked by a voice that was either disembodied or coming from a robot [13]. Females showed less social desirability scores with the disembodied voice, whereas males showed less social desirability with the robot. These findings suggest that males may have felt more open and honest with the robot than did females. In any case, these results indicate that males perceived the robot differently from females [13].

A study of proxemics, or the use of personal space and comfortable distances, involving robots examined personal preferences when a robot could approach a participant either directly from the front or at an angle from the side [14]. Results showed that although females were largely indifferent as to whether the robot approached from the front or side, males were much more uncomfortable when the robot approached from directly in front of them as opposed to the side [14]. Researchers suggested that a front approach may have been perceived as more combative by the males [14].

Other studies have looked more closely at opinions toward robots based on a person’s gender. The Negative Attitudes toward Robots Scale [15] has been used numerous times to show that females tend to have significantly stronger negative opinions toward robots than males [16-18]. One study found females had lower rates of robot liking and higher rates of *Robotphobia* than their male counterparts [19]. Researchers have also surveyed adult opinions on a mechanical robot at a public mall and found that females found the robot unpredictable, whereas males found the robot helpful [20]. Looking closer at helpfulness, another study found that males, regardless of age, rated a health care robot as more useful and were more hopeful for its future development than were their female counterparts, both before and after interacting with a health care robot [21].

Furthermore, it has been demonstrated that even the tendency to anthropomorphize itself can be impacted by gender [22]. Using 2 scales directly measuring a person’s tendency to anthropomorphize pets, gods, or artifacts, investigators found that females were more likely than males to anthropomorphize animals but found no differences in the tendency to anthropomorphize artifacts. However, in this study, the category of *artifacts* included both robots and mechanical devices, such as vehicles or computers, so no specific anthropomorphism rating for robots could be determined from this study.

### Purpose of the Study

The SEEK model represents a psychological theory of the determinants of anthropomorphism, which may have broader applicability to our understanding of why people make attributions of human like qualities to diverse nonhuman entities, including machines and robots. Preliminary support for the applicability of the SEEK model to the anthropomorphism of robots has already been provided, particularly as it relates to sociality and effectance motivation [5,23,24], and, to a lesser extent, elicited agent knowledge [12]. The primary purpose of this study was to further examine the SEEK factor of elicited agent knowledge by evaluating 2 specific hypotheses related to that factor.

#### Hypothesis 1

**Figure 1:**
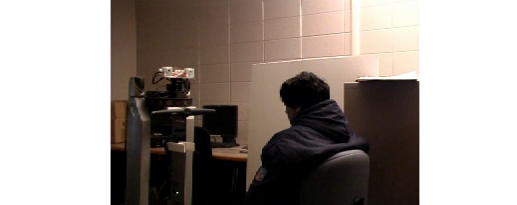
A screen capture from the experiment video of the nonhumanoid robot in dialogue with confederate.

**Figure 2:**
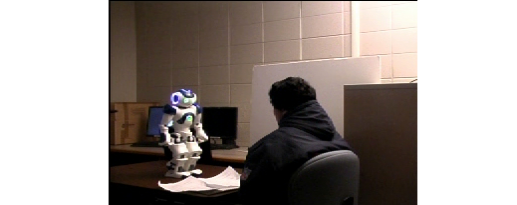
A screen capture from the experiment video of the humanoid robot in dialogue with confederate.

Participants should engage in more anthropomorphism toward a humanoid robot than toward one that is nonhumanoid because greater similarity of appearance to a person should allow participants to bring more self-knowledge to bear on their understanding of and attributions toward the humanoid agent. To test this hypothesis, we employed an *appearance* manipulation involving 2 different robots: one robot having a distinctly humanoid form, whereas the other clearly having a much less human like appearance (see Figures 1 and 2).

#### Hypothesis 2

Participants should anthropomorphize an autonomous robot more than a nonautonomous robot because of the greater ease with which autonomy allows humans to apply their own anthropocentric knowledge as a means of understanding the autonomous nonhuman agent’s behavior. We tested this hypothesis by using an *agency* manipulation to induce different beliefs about either the humanoid or nonhumanoid robots. One belief was that both robots were fully autonomous and capable of acting on their own volition, whereas the second belief was that an experimenter in another room was controlling the robots. This *agency* manipulation was intended to provide participants with distinctively different explanations for the behavior of the robot to which they were exposed. We expected participants to identify more closely with the autonomous agent, regardless of its appearance.

As noted above, females have a more negative view of robots than males [15-17] and have a greater tendency than males to anthropomorphize animals [22]. However, the implications of these findings for gender-based differences in anthropomorphism of robots are not clear. Thus, a secondary purpose of this study was to include gender as an additional factor in the evaluation of both hypotheses 1 and 2.

## Methods

### Participants

Participants consisted of 99 undergraduate students, 52 males and 47 females, between the ages of 18 and 22 years, enrolled at a midsized, private, Midwestern university. Participants voluntarily chose to be in this study and received course credit for their participation. We treated participants in accordance with the ethical standards of the American Psychological Association and the institutional review board approved the research protocol.

### Design

The design of this study conformed to a 2 (appearance: humanoid vs nonhumanoid) × 2 (agency: autonomous vs nonautonomous) × 2 (participant gender: male vs female) factorial design, with all factors varied between subjects. Participants were assigned randomly to each of the 4 groups.

### Materials

The following materials used in this study are organized according to whether they were administered before the experimental manipulations (pretest materials), during the manipulations themselves (experimental materials), or after the manipulations (posttest materials).

#### Pretest Materials

Several pretest scales were used to verify that our 4 groups did not differ on factors that could influence the results other than the explicitly manipulated factors. A total of 3 of the pretest scales, Desire for Control [25], University of California, Los Angeles (UCLA) Loneliness [26], and Need for Cognition [27] were used because they have been directly tied to the factors involved in the SEEK model [1,2]. Desire for Control and Loneliness are relevant to the SEEK factors of effectance motivation and sociality motivation, whereas Need for Cognition is linked to one’s ability to employ elicited agent knowledge [1,2]. By comparing the groups on these scales, we could verify that our independent groups in this study were not different in these SEEK related factors before experiencing our experimental manipulations. A shortened version of the Marlowe-Crowne scale [28,29], a questionnaire measuring social desirability, was included as a pretest check that our groups also did not differ in social desirability, which could influence their responses to the questionnaires used in this study.

#### Experimental Materials

During the experimental phase of this study, each participant was exposed to 1 agency manipulation story (either autonomous or nonautonomous) and 1 robot interaction video (depicting either the humanoid or nonhumanoid robot). During the course of the experiment, participants made no direct contact with either robot. All experimental materials listed below can be found in Multimedia Appendix 1.

##### Agency Manipulation Stories

The agency manipulation (autonomous vs nonautonomous) used in this study was delivered via 1 of 2 different stories read to participants just before they saw their designated humanoid or nonhumanoid robot video (see Multimedia Appendix 1). Both videos depicted the 2 morphologically distinct robots doing exactly the same things. The autonomous story said that the robot had artificial intelligence and the capability to perform fully autonomous behavior. In contrast, for the nonautonomous condition, the story explained that a human controlled the robot from another room.

##### Appearance Manipulation Videos

On the basis of previous work using virtual reality environments indicating that human evaluations of virtual robots are comparable in many respects with those obtained from observing similar physically present robots [30,31], along with the work reported by another study showing that measures taken via live interactions with a robot are comparable with those from video based interactions [32], we believed that video exposure to robots would produce effects comparable with those obtained from direct, physical exposure. Therefore, our appearance manipulation involved having participants view a brief video of a confederate experimenter interacting with either the humanoid or a nonhumanoid robot, depending on the appearance condition to which participants were assigned (see Multimedia Appendices 2 and 3).

The nonhumanoid robot was a PeopleBot, obtained from MobileRobots Inc (see Figure 1 and Multimedia Appendix 3). The nonhumanoid robot was approximately 5 feet tall and had small speakers sitting on either side of the upper shelf under the cameras and slightly elongated grippers to provide the impression of arm like appendages. The humanoid robot was a Nao Academics Edition, Version 3 Plus obtained from Aldebaran Robotics (see Figure 2 and Multimedia Appendix 2), which was approximately 1.9 feet tall weighing approximately 9.5 lbs.

Drawing on previous work showing humans prefer to interact with telepresence robots at eye level [33], the robots in this study were oriented such that the tops of their heads were near the top of the camera frame, making the heads of both equidistant from the floor. This resulted in positioning the confederate’s head and gaze at approximately the same viewing angle for both robots. To do this, the Nao robot stood on a table, whereas the PeopleBot remained on the floor, and the confederate remained seated in both videos (see Figures 1 and 2). Consistent with the findings of a longitudinal study by of HRI [34], we believed this arrangement would allow participants to respond more to robot appearance than to robot height. In addition, differences of up to 0.2 meters (0.7 ft) in robot height do not significantly influence opinions toward or comfort with robots [35,36].

The same script was used to create the 2 videos depicting identical interactions of a student with either the humanoid or nonhumanoid robot. The script depicts a conversation between the robot and a student in which the robot described and demonstrated some of its capabilities and then engaged the student in a brief discussion about college football. All robot movements were carefully selected so as to be comparable between the 2 robot platforms, and the same voice was used for both robots. The entire video lasted approximately 3.5 min.

#### Posttest Materials

Our posttest measures involved published scales previously used to determine how subjects perceived and anthropomorphized the robot they saw in the video.

##### Perception of Humanoid Robots Scale

The perception of humanoid robots’ scale, known as Perception of Humanoid Robots Scale (PERNOD) [37], was employed in this study as an appearance manipulation check to see how similar or different our participants viewed the humanoid and nonhumanoid robots we used. The PERNOD evaluates a participant’s perception of a particular robot on 6 separate dimensions: *graceful* related to the quickness or slowness with which it moved; *expressive* related to how the robot communicates emotion or friendliness; *useful* related to potential utility of the robot for humans; *controllable* related to how subservient to humans it appears; *durable*, which reflects a lack of concern about fragility or breakability of the robot; and *smooth*, which refers to the look or physical appearance of the platform being not angular or coarse. A 7-point scale was used for all items, and the scoring was such that higher values indicate stronger alignment with a dimension.

##### Epley et al Anthropomorphic Items

One measure of anthropomorphism used in this study was derived from several items used in a study with pet owners [2]. These measures consisted of 7 anthropomorphic (thoughtful, considerate, sympathetic, embarrassable, creative, devious, and jealous) and 7 behavioral (aggressive, agile, active, energetic, fearful, lethargic, and muscular) trait adjectives that participants were asked to rank from 1 to 14 in order of decreasing applicability to the robot they saw in the video. Separate sums of ranks were computed for both anthropomorphic and behavioral trait adjective categories for each participant. The scores were then reverse coded such that a higher sum of ranks signified that the adjectives in that category were rated as more applicable to the robot. These 2 groups of anthropomorphic and behavioral traits were created by Epley et al, and we retained this categorization for this study [2].

##### Fussel Adjective Checklist

A second measure of anthropomorphism used in this study was based on an adjective checklist used in an earlier study [38]. This checklist consisted of 40 adjectives, 10 in each of 4 categories: human sociability, other human, robotic, and false fillers [38]. There were both positive and negative adjectives in both human categories. A total of 2 of the other human adjectives were gender related and were separated, whereas the remaining 8 referred to what can be called *human personality* traits. A third category pertained to characteristics of robots, which itself can be subdivided into characteristics that clearly were mechanical and those that were not. The fourth category consisted of characteristics very likely to be rated as false for both humans and robots but which could not be subdivided in any obvious way. Our revised breakdown of the original 40-item [38] adjective checklist is shown in Table 1. It is important to note here that the adjectives in this table are exactly the same as those employed in the reference study, only their organization has been changed to distinguish positive or negative and mechanical or nonmechanical subcategories. Subjects designated each adjective as either true or false for the robot (humanoid or nonhumanoid) they saw.

For all of the categories in Table 1, except Gender, we computed a proportion of true responses across the adjectives in that category for each subject. This resulted in separate proportions for each subject for the categories of human social positive, human social negative, human personality positive, human personality negative, robotic mechanical, robotic nonmechanical, and false fillers. The 2 gender categories were mutually exclusive such that participants assigned the 1 robot they saw either a male or a female designation.

**Table 1:** The reorganized Fussel adjective checklist.

Category	Adjectives
Human social positive	Friendly, polite, sensitive, caring, and sociable
Human social negative	Rude, obnoxious, cold, impatient, and aggressive
Human personality positive	Organized, thorough, curious, and persistent
Human personality negative	Nervous, distractible, shallow, and disorganized
Gender	Male and female
Robotic nonmechanical	Android, artificial, automaton, mechanical, controllable, and robotic
Robotic mechanical	Synthetic, breakable, software, and portable
False fillers	Animal, wooden, wet, smelly, tubular, ceramic, cotton, striped, roasted, and bloody

##### Anthropomorphic Tendencies Scale

A third measure of anthropomorphism was the Anthropomorphic Tendencies Scale (ATS) [39]. The ATS measured 4 subscale dimensions of anthropomorphism: extreme anthropomorphism (the attribution of human like qualities to physical objects such as backpacks or cars), anthropomorphism toward pets, anthropomorphism toward gods or deities, and negative anthropomorphism, which reflects a tendency to display negative emotions toward physical objects such as cars or computers. These dimensions were rated on a Likert scale ranging from 1 (strongly disagree) to 5 (strongly agree). Mean scores that were greater than 4 represented agreement, means scores of 2 and below represented disagreement, whereas mean scores of 3 reflected a neutral rating. Although this scale may measure relatively stable human traits, it was used in this study as a dependent variable to see if ATS tendencies were influenced in any way by a particular combination of our explicitly manipulated independent variables (IVs, appearance and agency) or by participant gender.

### Procedure

The study was completed in 1 experimental session, following the sequence of the materials listed above: pretest, experimental manipulation, and posttest. In order, the pretest measures were the Desirability of Control Scale, the UCLA Loneliness Scale, the Need for Cognition Scale, and the Marlowe-Crowne Social Desirability scale. After the pretest measures, the experimenter read participants either the autonomous or nonautonomous story, depending on the agency manipulation condition to which the participant was assigned. Then, participants watched 1 of 2 videos, depending on their appearance manipulation condition, showing either a humanoid or a nonhumanoid robot interacting with a confederate actor. Finally, participants completed the posttest anthropomorphism measures, which, in order, were the Fussel adjective checklist, the PERNOD scale, the Epley et al anthropomorphic items, and finally the ATS. Upon completion of the third phase, the experimenter fully debriefed the participants before dismissal.

### Data Reduction and Analyses

All scales and measures used in this study were scored for individual participants following the procedures described in the articles in which they were originally published. All dependent variables reported in this study were examined with the Shapiro-Wilk test to verify they were normally distributed within each of the 4 separate groups formed by the 2 manipulated IVs, appearance and agency. Accordingly, parametric tests were used for the analysis of the data collected in this study, as they are the most powerful means to assess the effects of the IVs [40]. For these analyses, both appearance and agency IVs were treated as between subject factors. Participant gender was also included in these analyses as a third, between-subjects factor to determine how the IVs affected both males and females in our study. Therefore, each analysis conformed to a 2 (appearance: humanoid vs nonhumanoid) × 2 (agency: autonomous vs nonautonomous) × 2 (participant gender: male vs female) analysis of variance (ANOVA) plan. Effects were considered significant in any ANOVA with *P* values ≤.05. Effect sizes were calculated in all ANOVAs as partial eta squared (η^2^_p_) to determine the degree of association between the variables. Partial eta squared values between 0.01 and 0.06 are considered small effects, between 0.06 and 0.14 are considered medium effects, and above 0.14 are large effects [34]. All significant interactions in the ANOVAs were followed up with individual group comparisons, and the Bonferroni procedure was applied to correct for multiple comparisons.

## Results

### Pretest Measure Analyses

Means, SDs, and group size for each of the separate groups in this study are provided in Table 2 for all 4 of the pretest measures. Separate 2 (appearance) × 2 (agency) × 2 (gender) preliminary analyses were conducted for each pretest measure to determine if there were any initial differences among groups on Desire for Control, Loneliness, Need for Cognition, or the Marlowe-Crowne scales. Results indicated no significant main effects or interactions for any of the pretest measures, with the exception of a main effect of gender within the Desire for Control Scale, *F*_1,91_=5.62, *P*=.019, η^2^_p_=0.06, with males showing a greater desire for control than females, a finding consistent with the original work of Burger and Cooper [25].

**Table 2:** Means, SDs, and group size for each of the groups in this study for all 4 of the pretest measures employed.

Appearance, agency, and gender subgroups			Desire for Control, mean (SD)	Loneliness, mean (SD)	Need for Cognition, mean (SD)	Marlowe-Crowne, mean (SD)
Appearance	Agency	Gender (N)				
Humanoid	Autonomous	Male (13)	105.7 (9.1)	38.3 (6.7)	113.1 (26.1)	4.8 (2.9)
Humanoid	Autonomous	Female (12)	101.0 (12.5)	39.8 (6.2)	116.1 (12.0)	4.5 (2.5)
Humanoid	Nonautonomous	Male (12)	103.3 (12.2)	37.8 (6.2)	106.3 (28.8)	5.2 (2.7)
Humanoid	Nonautonomous	Female (13)	97.5 (11.4)	39.2 (8.1)	101.8 (25.6)	5.8 (2.9)
Nonhumanoid	Autonomous	Male (13)	99.6 (13.8)	39.8 (6.7)	112.4 (19.9)	3.8 (1.7)
Nonhumanoid	Autonomous	Female (12)	96.7 (9.6)	39.1 (5.4)	110.4 (16.0)	4.1 (1.8)
Nonhumanoid	Nonautonomous	Male (14)	103.0 (10.3)	37.4 (9.5)	112.3 (21.1)	5.7 (3.4)
Nonhumanoid	Nonautonomous	Female (10)	95.3 (8.1)	39.7 (9.2)	102.5 (9.8)	3.4 (2.8)

#### Perception of the Humanoid and Nonhumanoid Robots

**Figure 3:**
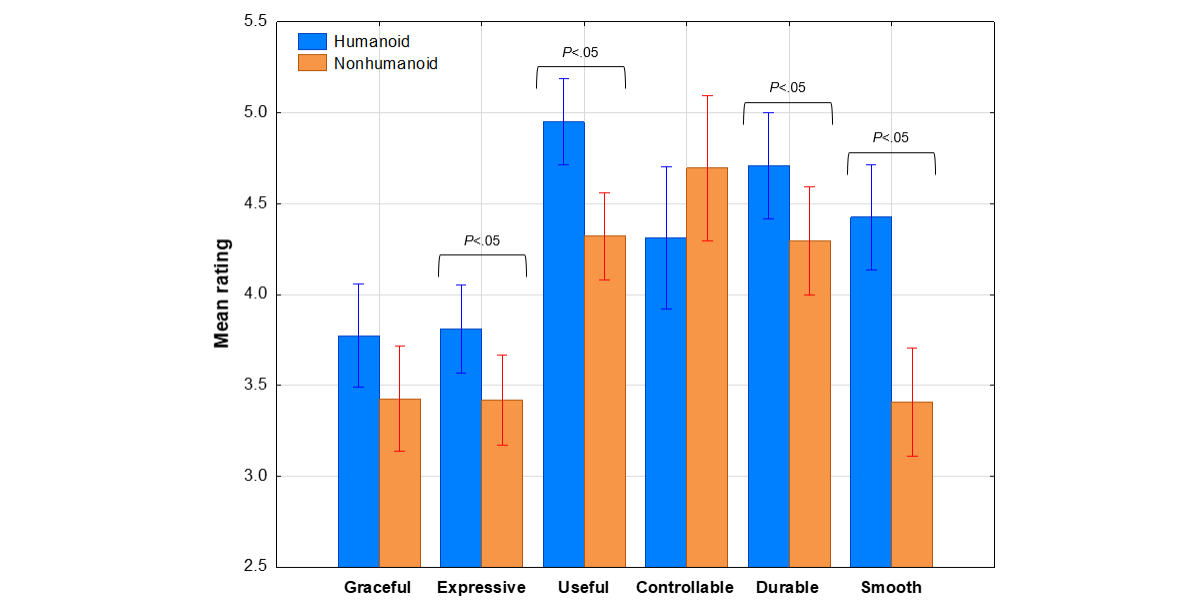
Mean rating on each perception to humanoid subscale as a function of robot appearance. Bars represent SE of the means.

Figure 3 shows the mean rating on each of the 6 PERNOD subscales as a function of robot appearance (humanoid vs nonhumanoid). As is evident from this graph, the humanoid robot was perceived differently than the nonhumanoid on all dimensions. Generally, the humanoid morphology was associated with higher ratings on all subscales except *controllable*, where the opposite was true.

The visual impressions in Figure 3 were confirmed by the results of an appearance × agency × gender multivariate analysis of variance (MANOVA) in which the 6 subscales were treated as multiple dependent measures. The main effect of appearance was significant in this analysis with a large effect size, *F*_6,86_=6.59, *P*<.001, η^2^_p_=0.31. Separate follow up appearance × agency × gender ANOVAs were performed to see if the appearance effect was significant for each subscale. The results of these tests revealed that only appearance effects were significant for the *expressive* (*F*_1,91_=5.9; *P*=.03), *useful* (*F*_1,91_=13.75; *P*<.001), *durable* (*F*_1,91_=3.83; *P*=.05), and *smooth* (*F*_1,91_=23.78; *P*<.001) subscales, marginally significant for the *graceful* subscale (*P*=.09) but not significant for the *controllable* subscale. No other main effects or interactions were significant. These findings indicate that participants did perceive the humanoid and nonhumanoid robots differently, as expected, which confirms the effectiveness of our appearance manipulation.

### Measures of Anthropomorphism

#### The Epley et al Adjectives

**Figure 4:**
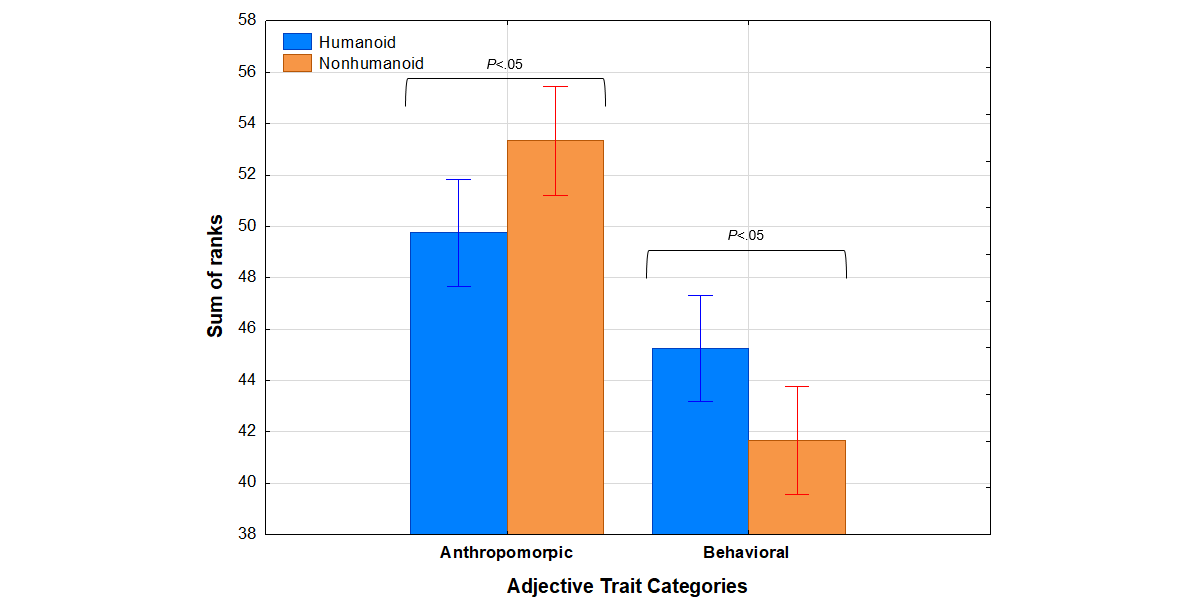
Mean rank sums for each category of Epley trait adjectives as a function of robot appearance. A higher sum of ranks indicates more applicable traits. Bars represent SE of the means.

Figure 4 depicts the reversed mean rank sums for each category of traits as a function of robot appearance. As is evident in Figure 4, generally higher rank sums for anthropomorphic than for behavioral traits were observed, meaning that participants believed that anthropomorphic traits were generally more applicable to both types of robots than were behavioral traits. However, within each trait category, appearance made a difference. Anthropomorphic traits were ranked higher for (were more applicable to) the nonhumanoid robot, whereas the behavioral traits were ranked higher for (were more applicable to) the humanoid robot.

Statistical confirmation for these observations was provided by an appearance × agency × gender × trait category ANOVA, which revealed a significant main effect of trait category, *F*_1,91_=29.45, *P*<.001, η^2^_p_=0.24, as well as an appearance × category interaction, *F*_1,91_=5.76, *P*=.02, η^2^_p_=0.06. Separate follow up appearance × agency × gender ANOVAs for each trait category revealed that the main effect of appearance was significant for both anthropomorphic *F*_1,91_=5.78, *P*=.02 and behavioral *F*_1,91_=5.76, *P*=.02 trait categories. For these analyses, neither gender nor agency were significant factors. These findings indicate that, contrary to our expectations, participants in this study were more willing to attribute anthropomorphic (ie, human like) traits to the nonhumanoid robot.

#### The Fussel Adjective Checklist

Due to the multiple categories within the Fussel adjective checklist, statistical information is provided below for each category. These categories are as follows: human adjectives, robotic adjectives, and false filler and gender adjectives.

##### Human Adjectives

As shown in Table 1, the human adjectives were organized into 2 main categories (social and personality), each with a positive and negative subdivision. To examine the effects of our IVs on these 4 human adjective categories and subcategories, we applied an appearance × agency × participant gender × category (social vs personality) × valence (positive vs negative) ANOVA to the proportions of true adjectives in each category. For this analysis, category and valence were both within subject factors. This overall analysis revealed only a significant main effect for agency, *F*_1,91_=4.88, *P*=.03, η^2^_p_=0.05, along with a significant category × valence interaction, *F*_1,91_=50.57, *P*<.001, η^2^_p_=0.37.

**Figure 5:**
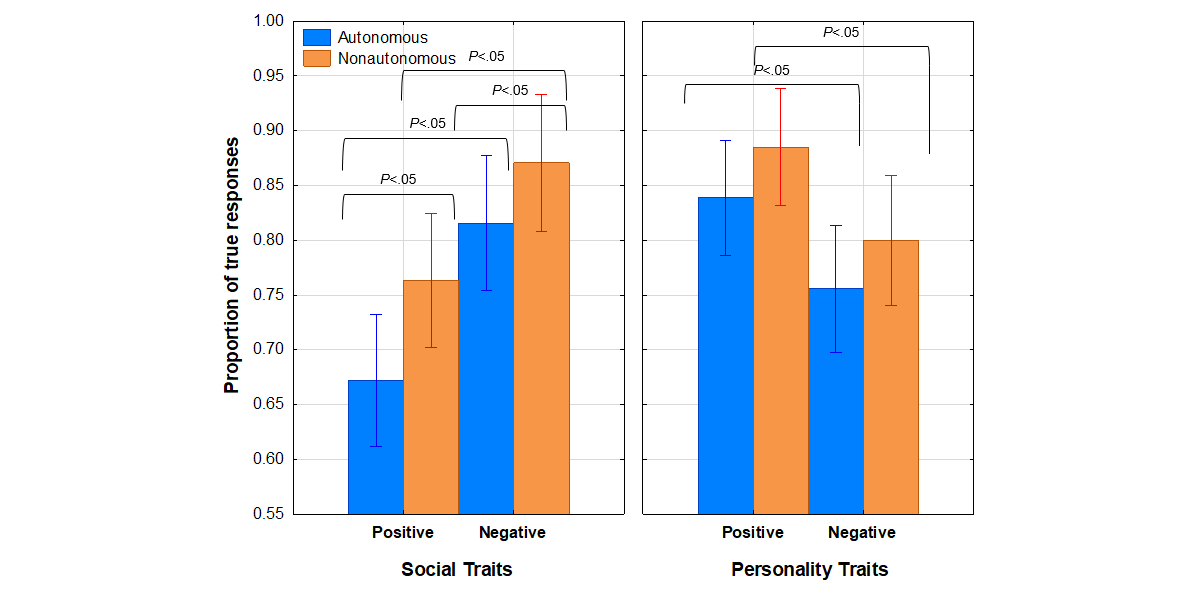
Mean proportion of true responses for Fussel human adjective categories as a function of agency (autonomous or nonautonomous), category (social or personality), and valence (positive or negative). Bars represent SE of the means.

Figure 5 shows the mean proportion of true responses as a function of agency, category, and valence. This graph illustrates that participants provided a higher proportion of true responses in each adjective category and subcategory for the nonautonomous robot. Moreover, it is clear that the category × valence interaction resulted from the reversal of the valence effect across categories. That is, for the 2 social adjective categories, participants ascribed more negative than positive attributes to the robot under both agency conditions, whereas the opposite was true for the 2 personality adjective categories.

To verify the basis for the interaction shown in Figure 5, appearance × agency × gender × valence ANOVAs were conducted separately for both social and personality adjective categories. For the social items analysis, the main effect of agency was significant, *F*_1,91_=5.30, *P*=.02, η^2^_p_=0.06, as was the main effect of valence (positive vs negative), *F*_1,91_=27.62, *P*<.001, η^2^_p_=0.23. For the personality items, only the main effect of valence was significant, *F* 1_,91_=7.35, *P*=.003, η^2^_*p*_=0.07. These outcomes verify that the valence effect was significant for both social and personality adjectives, but opposite in direction across categories. In addition, the effect of agency was arithmetically similar for both categories, but only reached significance for social adjectives. However, the fact that participants were more willing to attribute human like social qualities to the nonautonomous robot confirms that the agency manipulation was effective for this adjective category but also contradicts our original expectation that the autonomous robot condition would be perceived as the most human like.

##### Robotic Adjectives

**Figure 6:**
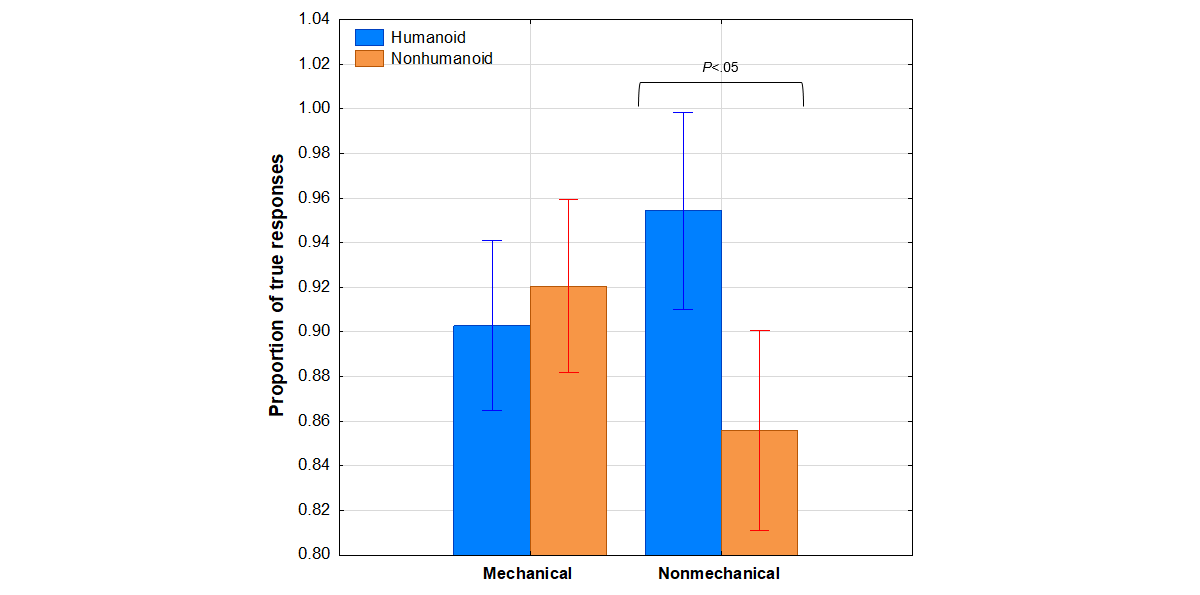
Mean proportion of true responses to the Fussel adjective checklist mechanical and nonmechanical robotic adjectives as a function of robot appearance. Bars represent SE of the means.

As shown in Table 1, the robotic adjectives from the original Fussel adjective checklist were subdivided into those that obviously referred to the mechanical characteristics of a robot and those that did not. Figure 6 shows the mean proportion of true responses to the mechanical and nonmechanical robotic adjectives as a function of robot appearance. What is apparent from this graph is that robot appearance made a difference only for the nonmechanical characteristics of the robotic adjectives.

The visual impressions evident in Figure 6 were confirmed by the results of an appearance × agency × participant gender × robotic adjective category (mechanical vs nonmechanical) ANOVA in which the appearance × robotic adjective category interaction was significant, *F*_1,91_=10.58, *P*=.001, η^2^_p_=0.10. Follow up tests showed that the interaction was because of a significant difference between appearance conditions only for the nonmechanical adjective category (*P*=.003). These results from the analysis of the robotic adjective category indicate that both robot morphologies were perceived to be equally mechanical, but the humanoid robot was perceived to be different from the nonhumanoid in nonmechanical ways (ie, portability).

##### False Filler and Gender Adjectives

The 2 remaining adjective categories in Table 1 were also examined. Participants in all conditions reported a low percentage of true responses for adjectives in the false filler category, which was expected based on the fact that these adjectives were selected specifically because they did not apply to humans or robots [38]. An appearance × agency × gender ANOVA of these items failed to reveal any significant effects. For the gender adjective category, a greater proportion of participants perceived the humanoid robot as male compared with the nonhumanoid robot, whereas the tendency to perceive either robot as female was equivalent for the 2 appearance categories. An analysis of the gender adjective proportions using an appearance × agency × participant gender × robot gender ANOVA revealed only a significant appearance × robot gender interaction, *F*_1,91_=5.83, *P*=.02, η2_p_=0.06. Follow up tests showed that the interaction was because of a significant difference between appearance conditions for the male robot gender category (*P*=.03), but not for the female category.

#### Anthropomorphic Tendencies Scale

**Figure 7:**
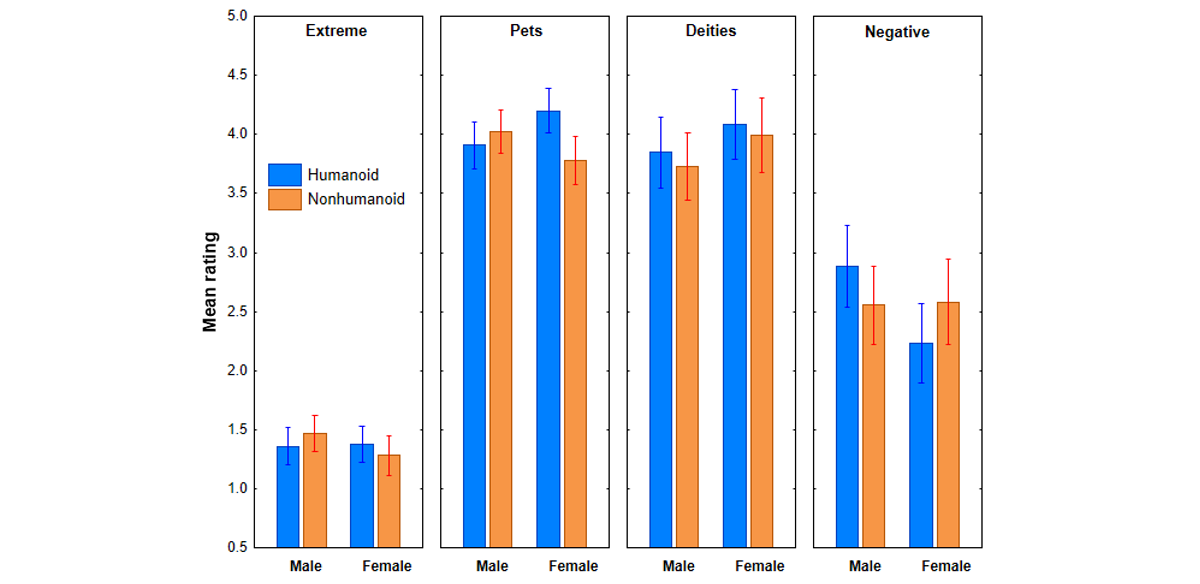
Mean ratings for each Anthropomorphic Tendencies Scale subscale as a function of robot appearance and participant gender. Bars represent SE of the means.

Figure 7 shows mean ratings for each ATS subscale as a function of robot appearance and participant gender. A total of 3 observations are apparent from this graph. First, participants generally agreed with statements reflecting anthropomorphism of pets and deities but disagreed with statements of extreme anthropomorphism. However, participants were more neutral about negative anthropomorphism statement. Second, ignoring robot appearance, male and female participants reported about the same levels of agreement with extreme anthropomorphism and anthropomorphism of pets but differed slightly in agreement with statements of anthropomorphism of deities and negative anthropomorphism. Third, the effect of robot appearance was different for males and females, most notably for anthropomorphism of pets and negative anthropomorphism.

To examine the trends in Figure 7, an appearance × agency × gender × ATS subscale (extreme vs pets vs deities vs negative) MANOVA was conducted, where the subscale scores were treated as separate dependent variables. This analysis revealed only a significant appearance × gender interaction, *F*_4,86_=4.10, *P*=.004, η^2^_p_=0.16. To better understand the appearance × gender interaction in the overall MANOVA, separate appearance × agency × gender interactions were conducted for each ATS subscale. The appearance × gender interactions were significant in these analyses only for anthropomorphism of pets, *F*_1,89_=7.47, *P*=.007, η^2^_p_=0.07 and negative anthropomorphism, *F*_1,89_=3.89, *P*=.05, η^2^_p_=0.04. Moreover, the main effect of gender was marginally significant for anthropomorphism of deities, *F*_1,89_=2.79, *P*=.09, η^2^_p_=0.03. Follow up individual group comparisons revealed that females differed significantly in their reported anthropomorphism of pets as a function of robot appearance (*P*=.02), and males expressed significantly more negative anthropomorphism than females under the humanoid appearance condition (*P*<.001). These results indicate, once again, that robot appearance was an effective variable in this study, at least for female versus male expressions of anthropomorphism of pets and negative anthropomorphism.

## Discussion

We can summarize the results of this study in the context of our original study purposes and hypotheses.

### Hypothesis 1: The Effect of Humanoid Appearance on Robot Anthropomorphism

In accord with the SEEK model of anthropomorphism [1], we hypothesized that a robot having a more human like morphology would provoke greater availability and use of homocentric knowledge than a less human like robot, which in turn might lead participants to engage in more anthropomorphism toward the humanoid than the nonhumanoid robot. To test this possibility, we employed an *appearance* manipulation involving robots with either a humanoid or a nonhumanoid form. Participants were asked to observe a videotaped interaction between 1 of these robots and a human and then complete several different measures of anthropomorphism that have been used in previous studies.

Results with the PERNOD scale showed that participants perceived the humanoid robot to be significantly more useful, expressive, graceful, and durable than the nonhumanoid robot and marginally more pleasing in appearance. These results demonstrated that the appearance manipulation made a difference on this measure and may also support the first hypothesis to the extent that the PERNOD expressive subscale (ie, the extent to which the robot communicates emotion and/or friendliness) is an indicator of anthropomorphic attributions. Of course, these findings do not tell us specifically which aspects of appearance were responsible for the differences revealed by this measure. Additional research will be needed to expand on these findings.

However, findings from the Epley and Fussel adjective measures of anthropomorphism [2,38] do not seem to support hypothesis 1. Although they revealed significant effects of the robot appearance factor, those effects were opposite in direction to our expectations. In both cases, participants were more willing to attribute human like traits to the nonhumanoid robot than to its humanoid counterpart.

It is not clear how to interpret our findings with the ATS in relation to hypothesis 1. These results were complicated by an appearance × gender interaction in which only females reported more anthropomorphism of pets under the humanoid appearance condition. In addition, females reported less negative anthropomorphism than males under the humanoid condition. At best, the ATS offers only limited support for our original hypothesis that the humanoid robot should provoke more anthropomorphic tendencies in our participants as the significant effects of appearance we observed with this measure were only for females.

### Hypothesis 2: The Effect of Agency on Robot Anthropomorphism

Our second hypothesis was that participants would anthropomorphize an autonomous robot more than one that was not autonomous. This expectation, derived from the SEEK model, was based on the idea that autonomy would allow participants to better understand and explain the robot’s behavior by applying their own anthropocentric knowledge to it. To test this possibility, we employed an *agency* manipulation in which participants were told that the robot they were about to see in the video either was sophisticated and capable of acting on its own or was being controlled by someone in another room. In contrast to a straightforward explanation of the nonautonomous robot’s behavior as being remotely controlled, we expected that participants would be more inclined to interpret the autonomous robot’s actions in more human like terms (eg, being friendly or sociable).

Interestingly, however, the only significant effects of agency obtained in this study ran counter to our expectations. For the Fussel adjective checklist items [38], we found that participants were more likely to make attributions of both positive (eg, friendly and sociable) and negative (eg, rude and aggressive) human sociality traits to the nonautonomous rather than the autonomous robot, regardless of appearance. The same trend appeared in the attributions of personality traits (eg, organized and distractible) but these findings did not achieve statistical significance. Therefore, once again as with appearance, we are left to wonder why our agency manipulation did not work as expected.

The manipulations in this study of appearance and agency both seemed to have independent influences upon how participants perceived the robot to which they were exposed. However, contrary to expectations based on the elicited agent knowledge factor in the SEEK model of anthropomorphism, participants were less willing to make attributions of human like social or personality traits toward a robot when its morphology had more human like features but were more willing to make those attributions when they were told that the robot was being remotely controlled by a person rather than acting on its own. As these influences did not appear to interact, it is appropriate to consider separate explanations for these somewhat surprising effects of both our IVs.

#### The Overall Size Factor

One possible explanation of the unexpected effects of robot appearance in our study is based on the overall size differential between the two robots. As the Nao humanoid robot was physically smaller in stature than the PeopleBot nonhumanoid platform, it is possible that the propensity to make human like attributions could have been influenced by this factor. We took two precautions to mitigate the possible effects of size difference in this study. The first was to expose participants to only one of our robots using prerecorded videos rather than using actual physical exposure to the robots. On the basis of the findings of an earlier study [32], we expected video exposure to yield comparable effects with actual physical exposure, and, although not eliminating the perception of size, video exposure might also mitigate the perception of apparent size relative to actual exposure, especially when participants do not see a second robot to which they can compare the first. A second precaution, noted earlier in the appearance manipulation video section, was that we positioned both robots for filming so that their heads were approximately equidistant from the floor and approximately at the same viewing angle with respect to the confederate used in the video.

Despite these precautions, however, there is at least modest evidence that robot size registered with our participants. For example, the right-hand portion of Figure 5 reveals that the humanoid robot differed significantly from its nonhumanoid counterpart in terms of nonmechanical attributes such as portability, a finding that may be partly a reflection of overall size. In addition, there were significant differences shown in Figure 3 in favor of the humanoid robot being perceived as the more useful platform, which also might be at least partly size related. Perhaps these differences are a reflection of the fact that participants considered the humanoid Nao to be more toy like (as it was perceived as more portable and useful) than the nonhumanoid PeopleBot. Possibly, these impressions influenced anthropomorphism tendencies in our participants. Further work needs to build on previous anthropomorphism research [33,35,36,41-43] not only in exploring the possible effects of robot height but also examining overall size. Undoubtedly, these considerations will prove to be quite relevant to a fuller understanding of HRI.

#### Indirect Agency

A different possible explanation to account for our counterintuitive finding that a remote-controlled robot was perceived as more human like than an *autonomous* robot is that participants in the nonautonomous conditions in this study actually made attributions toward what might be called the *indirect agent*. As participants in this condition were told that a person was controlling the robot, it is very possible that they viewed the nonautonomous robot merely as a kind of interface for a remote controlling human agent. Thus, attributions of humanness directed at the robot really might have been intended for the person thought to be behind the machine.

Other work indirectly supports the notion of indirect agency by showing that children are more empathetic toward teleoperated robots [44], people feel more social presence with teleoperated robots [45], and people have identified a teleoperated search and rescue robot as being warmer, safer, and more attentive than an autonomous robot [46]. In this study, participants were more willing to make human like attributions of positive sociality (ie, friendly, sensitive, and caring) to the nonautonomous robot being remotely controlled by a human than to an autonomous robot supposedly acting on its own. Quite possibly, this means that participants were making these human-like attributions toward the operator behind the robot rather than toward the robot itself.

The use of remotely controlled or teleoperated robots is a common strategy in studies of HRI that has come to be known as the *Wizard of Oz* paradigm [6,47,48]. The possibility that, under these circumstances, the robot might be viewed by participants merely as a surrogate for the human behind the scenes is a feature of this paradigm that has not received much attention, largely because the existence of the *wizard* usually is hidden from participants. Nonetheless, it is clear that we need to have a better understanding of when and how participants look past the machines with which they interact to the people they think are controlling those machines, or maybe even to those they think created or programmed them.

### Gender Effects

A secondary purpose of this study was to examine how males and females reacted to the manipulations in this study and responded to the various scales employed to measure robot perception and anthropomorphism. However, very few participant gender differences were observed. This finding suggests that male and female participants in this study perceived and made attributions about the 2 robotic platforms in essentially the same way. However, there was limited evidence that females in the humanoid robot condition reported more anthropomorphism toward pets and less negative anthropomorphism than females in the nonhumanoid robot condition.

### Conclusions

This study clearly indicated that physical robot appearance makes a difference in how people perceive robot platforms. The Nao robot in this study was perceived as more useful, expressive, graceful, and durable, and possibly smoother than the PeopleBot. These perceptions are important to document and explore in relation to how humans interact with different robotic platforms as well as what preferences humans might exhibit for interacting with 1 platform over another. Moreover, the finding in this study that the Nao humanoid robot was perceived as more masculine than the PeopleBot also may prove important in situations where perceived robot gender can influence HRI. These findings also suggest that participants made indirect agency attributions to the humans operating behind the robot, a finding of potential widespread significance in HRI. The robustness and boundary conditions for such indirect attributions need to be further explored and better understood.

Finally, we wish to note that general theories of anthropomorphism, such as the SEEK model [1], need to be more fully explored and tested in the context of HRI. In this study, we tested only 1 factor in SEEK model, elicited agent knowledge, and obtained some unexpected findings. It is important to understand how anthropomorphizing robots may be similar to or different from the anthropomorphism of other nonhuman entities. This work, as well as that of other SEEK model studies [5,12,23,24], represents important initial steps toward this goal.

## Appendix

Multimedia Appendix 1 Both agency priming scripts.

Multimedia Appendix 2 Video of humanoid robot and confederate interaction.

Multimedia Appendix 3 Video of nonhumanoid robot and confederate interaction.

## Abbreviations

ANOVA: analysis of variance
ATS: Anthropomorphic Tendencies Scale
HRI: human robot interaction
IVs: independent variables
MANOVA: multivariate analysis of variance
PERNOD: Perception of Humanoid Robots Scale
SEEK: Sociality, Effectance, Elicited agent Knowledge
