# Supplementary material for: Anthropomorphism of Robots: Study of Appearance and Agency
Source: JMIR Hum Factors. 2019 May 10;6(2):e12629. doi: 10.2196/12629 (PMC6533876; doi:10.2196/12629)
Supplement: Multimedia Appendix 1 [file humanfactors_v6i2e12629_app1.docx]

**Agency Script**

**Autonomous:** You will now view a short video of an interaction between a robot and person. Please watch the robot carefully as you will be asking some questions about your impressions of its interaction with the person afterwards. The robot in this video is equipped with a state-of-the-art hardware and software capabilities developed by robotics engineers allowing it to respond autonomously to its environment, perform tasks, and carry on intelligent conversations with humans. No human intervention is necessary. Press the space bar to continue to the video.

**Non-Autonomous/External** **Control:**  You will now view a short video of an interaction between a robot and person. Please watch the robot carefully as you will be asking some questions about your impressions of its interaction with the person afterwards. The robot in this video is unable to respond autonomously to its environment, perform tasks, or carry on intelligent conversations with humans. Instead, it is strictly controlled by software where each of its actions is determined beforehand. Press the space bar to continue.
